# Association of rs2072446 in the *NGFR* gene with the risk of Alzheimer's disease and amyloid‐β deposition in the brain

**DOI:** 10.1111/cns.13965

**Published:** 2022-09-08

**Authors:** Chen‐Yang He, Zuo‐Teng Wang, Ying‐Ying Shen, An‐Yu Shi, Hui‐Yun Li, Dong‐Wan Chen, Gui‐Hua Zeng, Cheng‐Rong Tan, Jin‐Tai Yu, Fan Zeng, Yan‐Jiang Wang, Alzheimer's Disease Neuroimaging Initiative

**Affiliations:** ^1^ Department of Neurology and Centre for Clinical Neuroscience, Daping Hospital Third Military Medical University Chongqing China; ^2^ Department of Neurology The General Hospital of Western Theater Command Chengdu China; ^3^ Department of Neurology, Qingdao Municipal Hospital Qingdao University Qingdao China; ^4^ Chongqing Key Laboratory of Ageing and Brain Diseases Chongqing China; ^5^ Department of Neurology and Institute of Neurology, Huashan Hospital, State Key Laboratory of Medical Neurobiology and MOE Frontiers Center for Brain Science, Shanghai Medical College Fudan University Shanghai China; ^6^ The Institute of Brain and Intelligence Third Military Medical University Chongqing China; ^7^ State Key Laboratory of Trauma, Burn and Combined Injury, Daping Hospital Third Military Medical University Chongqing China

**Keywords:** ADNI, Alzheimer's disease, amyloid‐β, p75NTR, polymorphism

## Abstract

**Introduction and Aims:**

Alzheimer's disease (AD) is the most common form of dementia with a complex genetic background. The cause of sporadic AD (sAD) remains largely unknown. Increasing evidence shows that genetic variations play a crucial role in sAD. P75 neurotrophin receptor (p75NTR, encoded by *NGFR*) plays a critical role in the pathogenesis of AD. Yet, the relationship between *NGFR* gene polymorphisms and AD was less studied. This study aims to analyze the relationship of *NGFR* gene polymorphism with the risk of AD in the Chinese Han population and amyloid‐β deposition in the ADNI cohort.

**Methods:**

This case–control association study was conducted in a Chinese Han cohort consisting of 366 sporadic AD (sAD) patients and 390 age‐ and sex‐matched controls. Twelve tag‐SNPs were selected and genotyped with a multiplex polymerase chain reaction‐ligase detection reaction (PCR‐LDR) method. The associations between tag‐SNPs and the risk of AD were analyzed by logistic regression. Moreover, another cohort from the Alzheimer's Disease Neuroimaging Initiative (ADNI) database was included to examine the association of one tag‐SNP (rs2072446) with indicators of amyloid deposition. Kaplan–Meier survival analysis and Cox proportional hazards models were used to test the predictive abilities of rs2072446 genotypes for AD progression. The mediation effects of Aβ deposition on this association were subsequently tested by mediation analyses.

**Results:**

After multiple testing corrections, one tag‐SNP, rs2072446, was associated with an increased risk of sAD (additive model, OR = 1.79, *P*_*adjustment*_ = 0.0144). Analyses of the ADNI cohort showed that the minor allele (T) of rs2072446 was significantly associated with the heavier Aβ burden, which further contributed to an increased risk of AD progression in *APOE ε4* non‐carrier.

**Conclusion:**

Our study found that rs2072446 in *NGFR* is associated with both the risk of sAD in the Chinese Han population and the amyloid burden in the ADNI cohort, which reveals the role of p75NTR in AD from a genetic perspective.

## Graphical abstract

rs2072446 in the *NGFR* gene increases the risk of sAD in Chinese Han population and is also correlated with higher amyloid burden and an increased risk for AD progression in the *APOE ε4* non‐carriers from the ADNI cohort, which represents a novel AD risk locus.

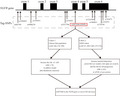

## 1 INTRODUCTION

Alzheimer's disease (AD) is the most common form of dementia that affects the aged population worldwide, with a complex genetic background.^1^ Despite the two characteristic pathological hallmarks of AD, senile plaques formed by amyloid‐β peptides (Aβ) and neurofibrillary tangles consisting of hyperphosphorylated tau protein have been well identified, its underlying mechanism remains elusive. Unlike early‐onset familial AD (fAD), which is mainly caused by mutations in genes coding amyloid precursor protein (APP) and its processing enzyme presenilin‐1 (PS1) and presenilin‐2 (PS2), the cause of sporadic AD (sAD) remains largely unknown.^2^ Increasing evidence shows that genetic variations play a crucial role in sAD.^1^, ^3^, ^4^ Genome‐wide association studies (GWAS) have identified more than 50 genetic risk factors associated with the disease, such as *APOE*, *SORL1, TREM2, CD33*, *ABCA7*, *MS4A6A*, and *CD2AP*.^2^ Aside from *APOE ε4* allele being the most acknowledged risk factor,^5^, ^6^ emerging single nucleotide polymorphisms (SNPs) involved in different pathways, including cholesterol metabolism, immunity, endocytosis, ubiquitination, and, more recently, Aβ clearance and tau biology, were revealed to be closely related to the risk of sAD.^2^, ^3^, ^7^ These findings strongly suggest the important role of genetic variations in the etiology of AD.

P75 neurotrophin receptor (p75NTR, encoded by the *NGFR* gene) is a low‐affinity receptor for almost all neurotrophins, and it has diverse functions in regulating neuronal growth, apoptosis, and synapse plasticity.^8^, ^9^ It is reported that p75NTR plays a critical role in the pathogenesis of AD, from metabolism and clearance of Aβ to mediating Aβ‐induced neuronal death, neurite degeneration, tau hyperphosphorylation, and cell cycle re‐entry.^9^ A recent study on the genome‐wide network‐based pathway analysis of CSF t‐tau/Aβ42 ratio in the Alzheimer's Disease Neuroimaging Initiative (ADNI) cohort further proved that *NGFR* was identified in the pathways of Aβ production and neurodegenerative diseases.^10^ Another study also discovered the association of genetic variations in *NGFR* with Aβ accumulation in 505 unrelated individuals enrolled in the Australian Imaging, Biomarker & Lifestyle (AIBL) study.^11^ However, there have been only two association studies on the relationship between *NGFR* gene polymorphism and the risk of AD to date, with conflicting results.^12^, ^13^ And both studies only selected certain specific SNPs which were unlikely to cover the entire gene. Given the important roles of p75NTR and genetic polymorphism in the pathogenesis of AD, it is necessary to further validate the relationship of *NGFR* gene polymorphism with AD.

## 2 METHODS

### 2.1 Study population

A total of 366 AD patients were consecutively recruited from the Registry of Neurodegeneration of Daping Hospital from January 2012 to December 2018, and 390 age‐ and sex‐matched controls were recruited from the hospital during the same period. The clinical assessment and diagnosis of AD dementia were performed following the protocol described in our previous studies.^14^ In brief, dementia was diagnosed based on criteria modified from the DSM‐IV. The subjects with dementia were further subjected to cranial CT or MRI. Diagnosis of probable AD was made according to the criteria of the National Institute of Neurological and Communicative Diseases and Stroke and the Alzheimer's Disease and Related Disorders Association (NINCDS–ADRDA). The demographic data, medical history, and cognitive and functional status were collected and assessed based on the formal questionnaire and a neuropsychological battery. These procedures were administered by trained interviewers composed of experienced neurologists.

All subjects enrolled in this study were ethnic Han people. The subjects were not eligible if they have: (1) a family history of dementia; (2) a concomitant neurologic disorder potentially affecting cognitive function (e.g., severe Parkinson's disease); (3) severe cardiac, pulmonary, hepatic, renal diseases or any kind of tumor; (4) declined to participate in the study. The study was approved by the Institutional Review Board of Daping Hospital and the procedures used in this study adhere to the tenets of the Declaration of Helsinki. Written consents for genetic screening were obtained from all participants or their legal representatives. Their confidentiality was preserved according to the guidelines for studies of human subjects.

### 2.2 Tag‐SNP selection and genotyping

The entire sequence of studied genes included the full length of the human *NGFR* gene plus 3 kb upstream and 1 kb downstream (23.728 kb in total). The genetic variation data of studied genes were obtained from the HapMap project (http://hapmap.ncbi.nlm.nih.gov/) for 45 unrelated Chinese Han people in Beijing (CHB), which was the latest data when this study was initiated. Eighteen SNPs with a minor allele frequency (MAF) ≥ 0.1 were firstly selected from NGFR. After converting the original data above to linkage format, we applied Haploview software (version 4.2) to choose tag‐SNPs with linkage disequilibrium (LD) threshold *r* ^2^ ≥ 0.8.^15^ In each LD block, priority was given to the tag‐SNP which was most frequently investigated in association studies with AD or was predicted to be of more important biological function by FASTSNP and SNPinfo^16^, ^17^ online software. The SNPs which did not form any LD blocks with others were also selected as tag‐SNPs to cover the entire gene as comprehensively as possible.

Venous blood was sampled and apportioned into sterile anti‐coagulation tubes. The genomic DNA was extracted using Wizard Genomic DNA Purification Kit (Promega, Madison) according to the product instruction. A multiplex polymerase chain reaction‐ligase detection reaction (PCR‐LDR) method was utilized for genotyping as described in our previous study.^14^ Briefly, for each SNP, the alleles were distinguished by different fluorescent labels of allele‐specific oligonucleotide probe pairs. Different SNPs were distinguished by distinct extended lengths at the 3′ end. The primers for the tag‐SNPs were shown in Table S1. *APOE* genotypes (determined by rs429358 and rs7412) were identified by the polymerase chain reaction‐restriction fragment length polymorphism (PCR‐RFLP) method as described in our previous study.^14^ The PCR primers are also shown in Table S1. The genotyping was carried out blindly to group status. A random sample accounting for approximately 5% (*n* = 38) of the total studied subjects was genotyped twice by different researchers for quality control, yielding a reproducibility of 100%.

### 2.3 About ADNI database

Alzheimer's disease neuroimaging initiative (including ADNI 1, ADNI 2, and ADNI Grand Opportunities [ADNI GO]) was a large repository of clinical and imaging data and can be accessed at http://adni.loni.usc.edu. All participants were recruited from more than 50 sites across the United States and Canada. The detailed criteria of exclusion and inclusion can be accessed at http://adni.loni.usc.edu/methods/documents/ (Procedures Manual). The ADNI embraced clinical and cognitive tests, cerebrospinal fluid and blood biomarkers, magnetic resonance imaging (MRI), amyloid PET, tau PET, and fludeoxyglucose PET. The procedures were described in previous studies.^18^, ^19^, ^20^, ^21^ Average AV45 standard uptake value ratios (SUVR) of frontal, anterior cingulate, precuneus, and parietal cortex relative to the cerebellum were utilized to assess brain Aβ burden. CSF Aβ42 was measured using the Elecsys β‐amyloid (1–42) CSF immunoassays on a Cobas E601 analyzer (software version 05.02). Given the varying levels of plasma Aβ42 across laboratories, only the latest samples measured by the Luminex immunoassay platform at the University of Pennsylvania were included. The proportion approach calculates the ratio between the volumes of interest (VOI) and total intracranial volume (ICV), producing a unitless value between 0 and 1. Further analyses, such as group comparisons, were carried out using this outcome measure. All parameters were available through the database website. The specific ADNI diagnostic criteria for distinguishing cognitively normal (CN), mild cognitive impairment (MCI), and AD participants were described previously.^22^, ^23^ ADNI samples were genotyped with the Human 610‐Quad BeadChip, Illumina Human Omni Express BeadChip, and Illumina Omni 2.5 M BeadChip. A total of 806 individuals (279 CN, 480 MCI, and 47 AD) from the ADNI database were included. In the longitudinal analyses, 276 CN and 469 MCI with follow‐up clinical data were included. One SNP in *NGFR*, rs2072446, was analyzed for this study. *APOE* and genome‐wide genotyping data were obtained from this database.

### 2.4 Statistical analysis

The age, as well as the proportion of sex and *APOE ε4* carriers of the two groups, were compared by *t*‐test and χ^2^‐test, respectively. The genotype distributions of each tag‐SNP in the control group were analyzed by χ^2^‐test for deviations from the Hardy–Weinberg equilibrium (HWE). The association between target SNPs and the risk of sAD with the adjustment for age, sex, and *APOE ε4* status was analyzed by unconditional Logistic regression, and five genetic models including codominant, dominant, recessive, over‐dominant, and additive models were applied. The statistical analysis was carried out by PASW version 18.0 for windows (SPSS, Inc.) and SNPStats online software.^24^ The statistical power of the case–control dataset was evaluated using Power and Sample Size software (version 3.0, http://biostat.mc.vanderbilt.edu/PowerSampleSize). All of the statistical tests were two‐sided, and *p* < 0.05 was defined as statistically significant. Bonferroni correction method was utilized for multiple testing.

As for data obtained from ADNI, all dependent variables (UC Berkeley‐AV45 PET, CSF Aβ42_,_ and plasma Aβ42) were normalized via “car” package of R software. Then these variables were standardized by z‐scale. Multiple linear regression models were run for each indicator of amyloid deposition (dependent variables) and rs2072446 (CC vs.TT/CT). Covariates include age (continuous), sex (male = 0, female = 1), *APOE ε4* status (non‐carrier = 0, carrier = 1), clinical diagnosis (CN = 0, MCI = 1, AD = 2), and ethnic category (Hispanic or Latino, not Hispanic or Latino, and unknown). In addition, we investigated the influence of age, sex, and *APOE ε4* on the association between rs2072446 and all indicators of amyloid deposition. Multiple linear regression was repeated including a 2‐way interaction term between rs2072446 and each of the three indicators as an additional independent variable. Subgroup analyses were performed stratified by *APOE ε4* status. Kaplan–Meier survival curves of AD progression (progress from CN or MCI to AD) were plotted based on rs2072446 genotypes (TT/CT vs. CC) stratified by *APOE ε4* carrier status. The log‐rank test was used to compare the survival distribution of subgroups with different rs2072446 genotypes. Cox proportional hazards models were used to test the predictive abilities of rs2072446 genotypes for AD progression. Moreover, mediation analyses were performed to test and quantify the mediation effects of Aβ deposition on the associations of rs2072446 genotypes and AD progression in *APOE ε4* non‐carriers (adjusted for age, sex, and education). Bootstrapping (10,000 iterations) methods were used to estimate the 95% CI. The “car,” “lm,” “glm,” “arm,” “survival,” “survminer,” “mediation,” and “ggplot2” packages in R 3.6.2 software were used to perform the above analyses.

## 3 RESULTS

### 3.1 Characteristics of the study population

Among the 366 sAD patients and 390 controls recruited, there were no significant differences in age (69.89 ± 9.67 vs. 68.69 ± 8.92, *p* = 0.077) and sex (female proportion: 53.8% vs. 49.0%, *p* = 0.18) between the two groups. As expected, more *APOE ε4*‐carriers (38.4% vs. 21.4%) were found in the sAD group (*p* = 3.80 × 10^−7^). The detailed characteristics of the study population were shown in Table S2.

### 3.2 Construction of LD blocks and selection of tag‐SNPs

**FIGURE 1:**
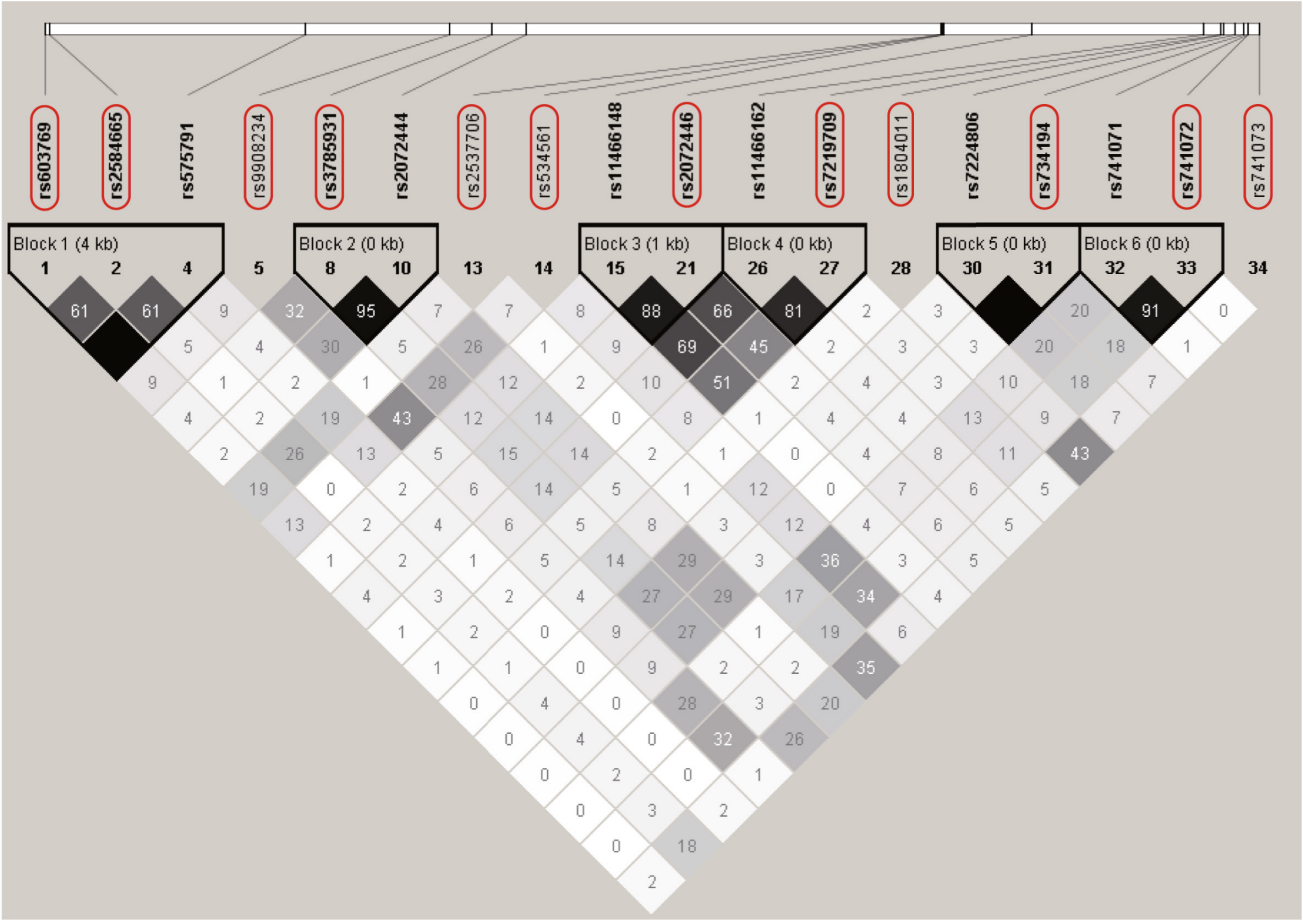
Construction of linkage disequilibrium blocks and selection of tag‐SNPs in the *NGFR* gene. The entire sequence of studied genes included the full length of the human *NGFR* gene plus 3 kb upstream and 1 kb downstream. The linkage disequilibrium (LD) plot was generated using genetic variation data from the HapMap project by Haploview software. Twelve tag‐SNPs within *NGFR* were finally selected (indicated by black rectangles). The level of pairwise r^2^ values indicating the correlation between every two SNPs was shown in grayscale (darker color indicates stronger correlation) with its value described as percentage in each cell.

According to the obtained SNP information and LD blocks constructed by Haploview software, 12 tag‐SNPs within *NGFR* were finally selected, which were located in the promoter (rs603769 and rs2584665), intron1 (rs9908234 and rs3785931), intron3 (rs2537706 and rs534561), exon4 (rs2072446) and exon6 (rs7219709, rs1804011, rs734194, rs741072, and rs741073), respectively. Each tag‐SNP and the SNPs in the same LD block are shown in Figure 1.

### 3.3 Allele frequencies and genotype distributions of the tag‐SNPs

The genotyping of 12 tag‐SNPs generated an average call rate of 99.66% in this study. The MAF of each tag‐SNP was similar to the data from the HapMap database (release #28), and genotype distributions of each SNP were in agreement with HWE (*p* > 0.05). The locus information, MAF, and HWE *p* values of each SNP are shown in S3.

### 3.4 Association between tag‐SNPs within the NGFR gene and the risk of sAD

Five genetic models, including codominant, dominant, recessive, over‐dominant and additive models were applied to assess the association between tag‐SNPs and the risk of sAD. As shown in Table 1, after adjustments for age, sex and *APOE* status, rs2584665 (codominant model, OR = 0.62, 95% CI = 0.43–0.88, *p* = 0.026; dominant model, OR = 0.64, 95% CI = 0.44–0.88, *p* = 0.0073; over‐dominant model, OR = 0.62, 95% CI = 0.44–0.89, *p* = 0.0082; additive model, OR = 0.67, 95% CI = 0.49–0.92, *p* = 0.012), rs9908234 (additive model, OR = 0.77, 95% CI = 0.61–0.98, *p* = 0.035), rs2537706 (dominant model, OR = 0.69, 95% CI = 0.49–0.97, *p* = 0.034; additive model, OR = 0.68, 95% CI = 0.49–0.93, *p* = 0.014), and rs741072 (over‐dominant model, OR = 0.73, 95% CI = 0.54–0.97, *p* = 0.033) was associated with a reduced risk of sAD; rs3785931 (codominant model, OR = 1.82, 95% CI = 1.19–2.79, *p* = 0.022; dominant model, OR = 1.46, 95% CI = 1.05–2.02, *p* = 0.023; recessive model, OR = 1.52, 95% CI = 1.05–2.19, *p* = 0.025; additive model, OR = 1.35, 95% CI = 1.09–1.67, *p* = 0.0056), rs7219709 (dominant model, OR = 1.49, 95% CI = 1.01–2.18, *p* = 0.041; additive model, OR = 1.47, 95% CI = 1.05–2.05, *p* = 0.024), and rs2072446 (codominant model, OR = 1.74, 95% CI = 1.17–2.59, *p* = 0.0051; dominant model, OR = 1.83, 95% CI = 1.24–2.70, *p* = 0.0022; over‐dominant model, OR = 1.70, 95% CI = 1.14–2.54, *p* = 0.0084; additive model, OR = 1.79, 95% CI = 1.25–2.56, *p* = 0.0012) was associated with an increased risk of sAD. However, all of these associations did not retain after Bonferroni correction except for those of rs2072446 in the dominant (*p* = 0.0264) and additive (*p* = 0.0144) models. The SNP rs2072446, which is located on exon 4 of *NGFR*, changes the 205th amino acid of p75NTR protein from serine to leucine, and has the most significant association with the risk of sAD (*P*_adjustment_ <0.05). It is predicted to be an exon splicing silencer (ESS) by SNPinfo,^17^ and not conserved which may affect the structure and function of p75NTR by SIFT^25^ and MutationTaster^26^ online software.

**TABLE 1:** Genotype distributions of the tag‐SNPs and their associations with the risk of sporadic Alzheimer's disease

Tag‐SNPs	Genotypes	Control group	sAD group	Genetic models^a^	OR value	95% CI	*p* Value^b^
rs603769	A/A	233 (60.2%)	239 (66.2%)	Codominant	0.72	0.53–1.00	0.14
	A/G	137 (35.4%)	105 (29.1%)		0.86	0.42–1.76	
	G/G	17 (4.4%)	17 (4.7%)	Dominant	0.74	0.54–1.00	0.052
				Recessive	0.96	0.47–1.95	0.90
				Over‐dominant	0.73	0.53–1.00	0.051
				Additive	0.8	0.62–1.04	0.095
rs2584665	A/A	277 (71.6%)	288 (79.6%)	Codominant	0.62	0.43–0.88	0.026
	A/C	104 (26.9%)	68 (18.8%)		0.73	0.23–2.35	
	C/C	6 (1.5%)	6 (1.6%)	Dominant	0.63	0.44–0.88	0.0073
				Recessive	0.81	0.25–2.62	0.73
				Over‐dominant	0.62	0.44–0.89	0.0082
				Additive	0.67	0.49–0.92	0.012
rs9908234	A/A	204 (52.6%)	217 (60.3%)	Codominant	0.81	0.59–1.10	0.099
	A/G	151 (38.9%)	124 (34.4%)		0.55	0.30–1.02	
	G/G	33 (8.5%)	19 (5.3%)	Dominant	0.76	0.57–1.03	0.072
				Recessive	0.60	0.33–1.10	0.092
				Over‐dominant	0.86	0.63–1.17	0.33
				Additive	0.77	0.61–0.98	0.035
rs3785931	T/T	126 (32.6%)	90 (24.9%)	Codominant	1.33	0.94–1.88	0.022
	T/C	194 (50.1%)	185 (51.3%)		1.82	1.19–2.79	
	C/C	67 (17.3%)	86 (23.8%)	Dominant	1.46	1.05–2.02	0.023
				Recessive	1.52	1.05–2.19	0.025
				Over‐dominant	1.04	0.77–1.39	0.8
				Additive	1.35	1.09–1.67	0.0056
rs2537706	G/G	273 (71.1%)	278 (77.4%)	Codominant	0.74	0.52–1.04	0.025
	G/A	102 (26.6%)	79 (22.0%)		0.21	0.05–1.02	
	A/A	9 (2.3%)	2 (0.6%)	Dominant	0.69	0.49–0.97	0.034
				Recessive	0.23	0.05–1.10	0.037
				Over‐dominant	0.76	0.53–1.07	0.11
				Additive	0.68	0.49–0.93	0.014
rs534561	C/C	180 (46.5%)	180 (49.7%)	Codominant	0.87	0.64–1.18	0.59
	C/G	174 (45.0%)	148 (40.9%)		1.07	0.63–1.83	
	G/G	33 (8.5%)	34 (9.4%)	Dominant	0.90	0.67–1.21	0.49
				Recessive	1.14	0.69–1.91	0.61
				Over‐dominant	0.86	0.64–1.16	0.32
				Additive	0.96	0.77–1.21	0.76
rs2072446	C/C	335 (86.6%)	278 (77.0%)	Codominant	1.74	1.17–2.59	0.0051
	C/T	50 (12.9%)	75 (20.8%)		4.01	0.82–19.62	
	T/T	2 (0.5%)	8 (2.2%)	**Dominant**	**1.83**	**1.24–2.70**	**0.0022**
				Recessive	3.63	0.74–17.76	0.080
				Over‐dominant	1.70	1.14–2.54	0.0084
				**Additive**	**1.79**	**1.25–2.56**	**0.0012**
rs7219709	C/C	330 (85.1%)	282 (77.9%)	Codominant	1.40	0.94–2.08	0.072
	C/T	54 (13.9%)	70 (19.3%)		2.65	0.81–8.68	
	T/T	4 (1.0%)	10 (2.8%)	Dominant	1.49	1.01–2.18	0.041
				Recessive	2.49	0.76–8.17	0.11
				Over‐dominant	1.37	0.92–2.04	0.12
				Additive	1.47	1.05–2.05	0.024
rs1804011	C/C	296 (77.1%)	279 (77.7%)	Codominant	0.95	0.66–1.37	0.91
	C/A	81 (21.1%)	73 (20.3%)		1.20	0.41–3.52	
	A/A	7 (1.8%)	7 (2.0%)	Dominant	0.97	0.68–1.38	0.88
				Recessive	0.67	0.25–1.78	0.41
				Over‐dominant	1.21	0.42–3.55	0.72
				Additive	0.99	0.73–1.36	0.97
rs734194	T/T	185 (47.7%)	199 (55.1%)	Codominant	0.78	0.58–1.07	0.16
	T/G	167 (43.0%)	137 (38.0%)		0.65	0.37–1.15	
	G/G	36 (9.3%)	25 (6.9%)	Dominant	0.76	0.57–1.02	0.069
				Recessive	0.73	0.42–1.25	0.25
				Over‐dominant	0.83	0.63–1.12	0.22
				Additive	0.80	0.63–1.01	0.055
rs741072	C/C	143 (36.9%)	156 (43.2%)	Codominant	0.72	0.53–0.99	0.10
	C/T	202 (52.1%)	158 (43.8%)		0.96	0.59–1.56	
	T/T	43 (11.0%)	47 (13.0%)	Dominant	0.76	0.56–1.03	0.076
				Recessive	1.15	0.73–1.81	0.55
				Over‐dominant	0.73	0.54–0.97	0.033
				Additive	0.89	0.71–1.11	0.31
rs741073	G/G	223 (57.5%)	224 (61.9%)	Codominant	0.85	0.62–1.16	0.54
	G/A	143 (36.8%)	120 (33.1%)		0.84	0.43–1.63	
	A/A	22 (5.7%)	18 (5.0%)	Dominant	0.84	0.63–1.14	0.27
				Recessive	0.90	0.47–1.72	0.74
				Over‐dominant	0.86	0.63–1.17	0.33
				Additive	0.88	0.69–1.12	0.3

*Note*: Figures in bold indicate the retained association after Bonferroni correction.

Abbreviations: *APOE*, apolipoprotein E gene; CI, confidence interval; OR, odds ratio.

^a^ Assuming M represents the major allele and m represents the minor allele, each genetic model can be described as follows: codominant: M/m versus M/M and m/m versus M/M, two OR values were listed from top to bottom in corresponding columns; dominant: (m/m + M/m) versus M/M; recessive: m/m versus (M/M + M/m); over‐dominant: M/m versus (M/M + m/m); additive: m/m and M/m were weighed 2 and 1 respectively to M/M. All models were adjusted by age, sex, and *APOE ε4* status.

^b^ The given *p* values were not corrected by Bonferroni correction.

### 3.5 Correlation between rs2072446 and Aβ deposition in ADNI cohort

A total of 806 individuals (279 CN, 480 MCI, and 47 AD) from the ADNI database were included (Table 2). No significant association was found between rs2072446 and amyloid burden in the total population (Table S4). However, as shown in Table 3, the interaction between rs2072446^TT/CT^ and *APOE ε4* was significant, indicating that *APOE ε4* status moderates the association between rs2072446 and amyloid deposition (AV45 PET: *β* = −0.7479, *p* = 0.0031; CSF Aβ42: *β* = 0.5086, *p* = 0.0403); meanwhile no interaction was not found between rs2072446^TT/CT^ and each of age and sex. In the subgroup of *APOE ε4* non‐carriers, the minor allele (T) of rs2072446 was significantly associated with amyloid deposition (AV45 PET: *β* = 0.6045, *p* = 0.0010; CSF Aβ42: *β* = −0.4433, *p* = 0.0118) (Table 4). No association was found between rs2072446 and other AD endophenotypes, including the CSF levels of tau, hyperphosphorylated tau, and normalized brain volume (Table S5).

**TABLE 2:** Characteristics of participants from the ADNI database

Characteristic	Total (*n* = 806)	CN (*n* = 279)	MCI (*n* = 480)	AD (*n* = 47)	*P* (CN vs. MCI)	*P* (MCI vs. AD)	*P* (CN vs. AD)
Age (years, mean ± SD)	73.26 ± 7.07	74.48 ± 5.57	72.34 ± 7.45	75.36 ± 9.27	<0.001	0.01	0.53
Sex (female, %)	360 (44.6)	144 (51.61)	198 (41.25)	18 (38.30)	0.01	0.70	0.09
Education (years, mean ± SD)	16.12 ± 2.76	16.42 ± 2.66	15.98 ± 2.86	15.72 ± 2.65	0.06	0.43	0.09
*APOE ε4* carriers (*n*, %)	328 (40.7)	76 (27.24)	218 (45.42)	34 (72.34)	<0.001	<0.001	<0.001
MMSE score (mean ± SD)	28.01 ± 2.073	29.07 ± 1.15	27.90 ± 1.68	22.85 ± 1.91	<0.001	<0.001	<0.001
ADAS11 score (mean ± SD)	8.82 ± 5.20	5.77 ± 2.94	9.54 ± 2.94	19.60 ± 6.71	<0.001	<0.001	<0.001
AV45	1.20 ± 0.23	1.12 ± 0.19	1.20 ± 0.23	1.39 ± 0.23	<0.001	<0.001	<0.001
CSF Aβ42 (pg/ml)	1060.82 ± 460.14	1223.38 ± 449.87	1013.42 ± 444.28	714.15 ± 349.54	<0.001	<0.001	<0.001
Plasma Aβ42 (pg/ml)	37.32 ± 12.11	38.45 ± 12.83	36.40 ± 11.45	NA	0.16	NA	NA
Entorhinal cortex/ICV	2.40e‐3 ± 4.69e‐4	2.53e‐3 ± 4.18e‐4	2.37e‐3 ± 4.71e‐4	1.93e‐3 ± 3.86e‐4	<0.001	<0.001	<0.001
Hippocampus/ICV	4.65e‐3 ± 7.78e‐4	4.90e‐3 ± 6.24e‐4	4.58e‐3 ± 8.05e‐4	3.83e‐3 ± 6.31e‐4	<0.001	<0.001	<0.001
Whole Brain/ICV	6.83e‐1 ± 4.81e‐2	6.85e‐1 ± 4.63e‐2	6.83e‐1 ± 4.98e‐2	6.69e‐1 ± 3.79e‐2	0.54	0.02	0.03

*Note*: Group comparison in continuous variables was performed using ANOVA and non‐parametric Kruskal–Wallis H test. Chi‐squared tests were used for categorical variables.

Abbreviations: AD, Alzheimer's disease; ADAS, Alzheimer's Disease Assessment Scale; *APOE*, apolipoprotein E gene; AV45,18F‐AV45 amyloid‐PET; Aβ, amyloid‐beta; CN, cognitively normal; CSF, cerebrospinal fluid; ICV, intracranial volume; MCI, mild cognitive impairment; MMSE, mini‐mental state exam; NA, not available; SD, standard deviation.

**TABLE 3:** Moderating effects of age, sex, and *APOE ε4* on the association between rs2072446 and indicators of amyloid deposition in the ADNI cohort

Variable or interaction	AV45		CSF Aβ42 (pg/ml)		Plasma Aβ42 (pg/ml)	
	*β*	*p* Value	*β*	*p* Value	*β*	*p* Value
Model for age effect						
rs2072446^TT/CT^	0.5517	0.6451	−0.9821	0.4120	0.3070	0.9197
rs2072446^TT/CT^ × age	−0.0045	0.7829	0.0106	0.5150	−0.0030	0.9403
Model for sex effect						
rs2072446^TT/CT^	−0.0301	0.9408	0.1673	0.6577	0.5870	0.3881
rs2072446^TT/CT^ × sex	0.1642	0.5119	−0.2556	0.2961	−0.3403	0.4312
Model for *APOE ε4* effect						
rs2072446^TT/CT^	0.5338	**0.0010**	−0.4155	**0.0087**	0.0855	0.7782
rs2072446^TT/CT^ × *APOE ε4*	−0.7479	**0.0031**	0.5086	**0.0403**	−0.0121	0.9778

*Note*: Adjusted for age, sex, *APOE ε4* status, clinical diagnosis, and ethnic category. Significant results (*p* value < 0.05) were indicated in bold.

Abbreviations: *APOE*, apolipoprotein E gene; AV45, 18F‐AV45 amyloid‐PET; Aβ, amyloid‐beta; CSF, cerebrospinal fluid.

**TABLE 4:** Associations of rs2072446 with indicators of amyloid deposition in the ADNI cohort stratified by *APOE ε4* status

SNP (Genotype)	AV45			CSF Aβ42			Plasma Aβ42		
	*n*	Mean ± SD	*β* *p* Value	*n*	Mean ± SD	*β* *p* Value	*n*	Mean ± SD	*β* *p* Value
*APOE ε4* carriers									
rs2072446									
CC	199	1.31 ± 0.22	−0.2089 0.3129	222	817.02 ± 389.76	0.1090 0.6058	95	34.18 ± 10.06	0.0766 0.8086
TT/CT	22	1.27 ± 0.28		23	854.87 ± 415.78		11	35.86 ± 7.72	
*APOE ε4* non‐carriers									
rs2072446									
CC	268	1.10 ± 0.18	**0.6045** **0.0010**	339	1237.63 ± 433.76	**−0.4433** **0.0118**	159	39.10 ± 12.90	0.0669 0.8300
TT/CT	32	1.23 ± 0.24		33	1028.05 ± 381.70		11	40.11 ± 15.47	

*Note*: Adjusted for age, sex, clinical diagnosis, and ethnic category. Significant differences after Bonferroni correction were indicated in bold.

Abbreviations: AD, Alzheimer'’s disease; *APOE*, apolipoprotein E gene; AV45, 18F‐AV45 amyloid‐PET; Aβ, amyloid‐beta; CSF, cerebrospinal fluid; SD, standard deviation; SNP, single nucleotide polymorphism.

**FIGURE 2:**
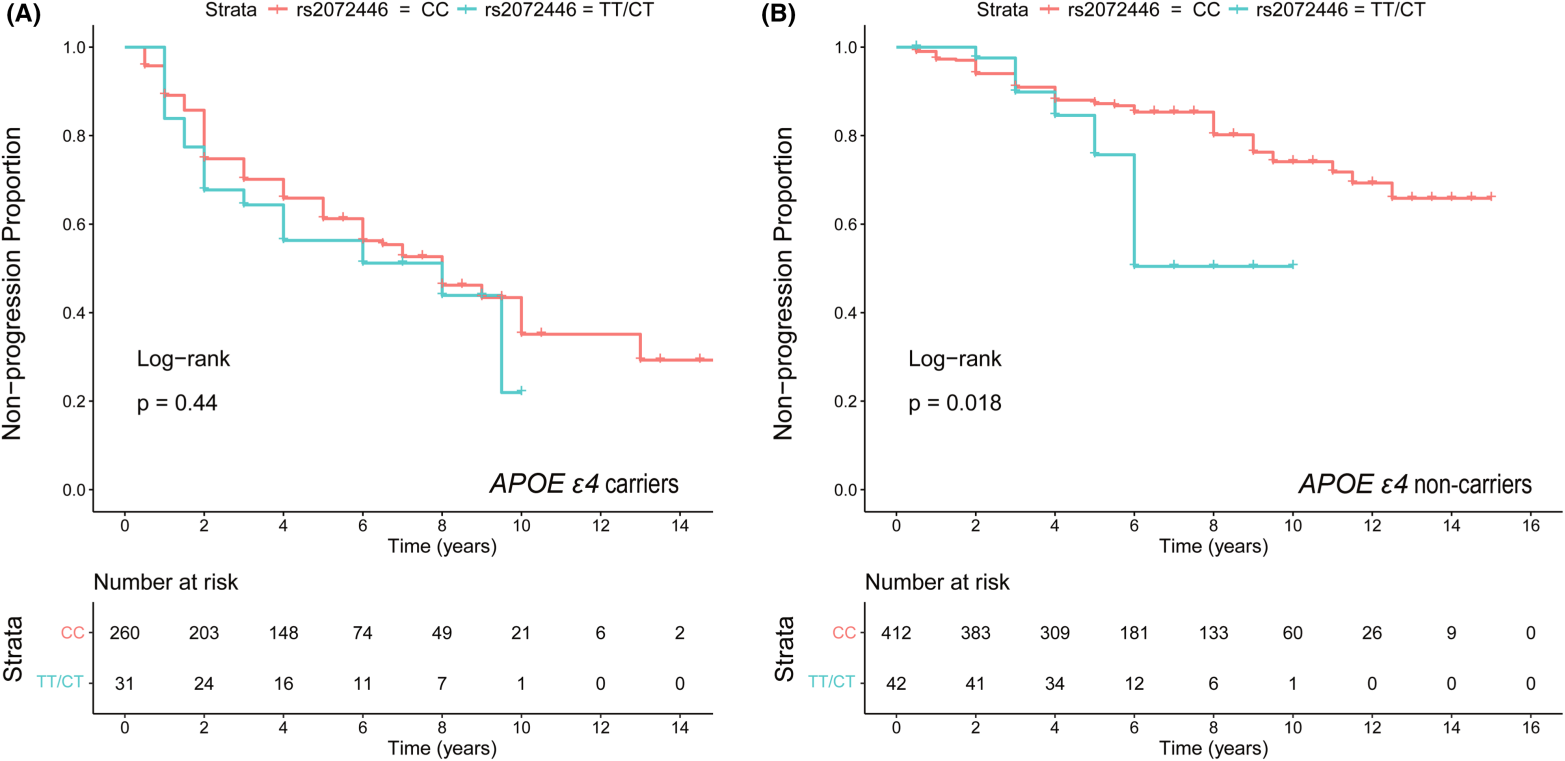
Associations of the rs2072446 genotypes with AD progression in the ADNI cohort. Kaplan–Meier survival analysis suggested that the rs2072446^TT/CT^ (plogrank = 0.016) was significantly associated with a shorter estimated time of AD progression in *APOE ε4* non‐carriers (B), but not in *APOE ε4* carriers (A).

**FIGURE 3:**
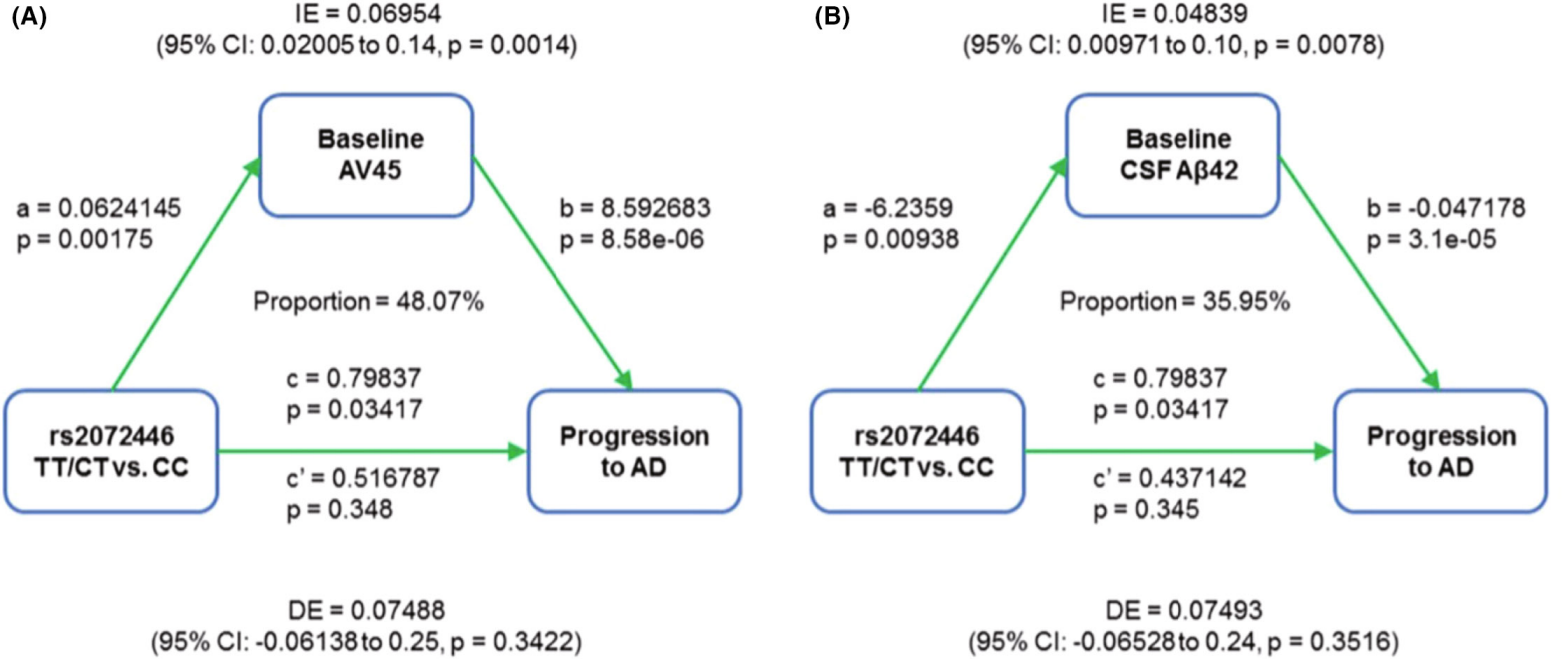
Mediation effects of baseline Aβ on the associations of rs2072446 genotypes with AD progression in *APOE ε4* non‐carriers from the ADNI cohort. After controlling for a range of potential confounders (age, sex, and years of education), the associations of rs2072446 with AD progression were mediated through baseline Aβ burden, which was 48.07% attributed to AV45 uptake (A) and 35.95% attributed to CSF Aβ42 (B).

In Kaplan–Meier survival analyses, rs2072446^TT/CT^ (plogrank = 0.018) was significantly associated with the shorter estimated time of AD progression in *APOE ε4* non‐carriers (Figure 2). Moreover, the rs2072446^TT/CT^ constituted a markedly increased risk for AD progression with an HR of 2.103 (95% CI = 1.0984–4.025; *p* = 0.0249) (Table 5). In the mediation analyses, the associations of rs2072446 with AD progression were mediated through baseline Aβ, which was 48.07% attributed to AV45 uptake, and 35.95% attributed to CSF Aβ42, respectively (Figure 3).

**TABLE 5:** Baseline demographic characteristics and rs2072446 genotypes as predictors of time to AD progression

Characteristic	*APOE ε4* carrier		*APOE ε4* non‐carrier	
	Hazard ratio (95% CI)	*p* Value	Hazard ratio (95% CI)	*p* Value
rs2072446 (TT/CT vs. CC)	0.9646 (0.5632–1.652)	0.8957	2.103 (1.0984–4.025)	**0.0249**
Age	1.0493 (1.0214–1.078)	**0.0005**	1.043 (1.0109–1.076)	**0.0084**
Sex (male vs. female)	0.7599 (0.516–1.119)	0.1643	1.262 (0.7869–2.025)	0.3339
Education	1.0116 (0.9498–1.077)	0.7196	1.024 (0.9466–1.109)	0.5499
Diagnosis (MCI vs. CN)	5.6428 (3.1733–10.034)	**<0.0001**	5.138 (2.8953–9.12)	**<0.0001**

*Note*: Cox proportional hazard models were used to assess the ability of demographic variables (rs2072446 genotype, age, diagnosis, and education) to predict clinical disease progression of AD over the 5.19 years (mean) follow‐up period.

Abbreviations: *APOE*, apolipoprotein E gene; CI, confidence intervals; CN, cognitively normal; MCI, mild cognitive impairment.

## 4 DISCUSSION

The present study mainly aims to investigate the association between genetic polymorphism of *NGFR* and the risk of Alzheimer's disease. To the best of our knowledge, it is by far the first study revealing that rs2072446 is associated with an increased risk of AD in the Chinese Han population. Moreover, rs2072446 is also associated with amyloid deposition and an increased risk of AD progression in *APOE ε4* non‐carriers in the cohort from the ADNI database.

P75NTR is a pan‐receptor for nerve growth factor (NGF) and other neurotrophins including brain‐derived neurotrophic factor (BDNF), neurotrophin‐3 (NT3), and neurotrophin‐4/5 (NT4/5).^27^, ^28^ Our previous study found that Aβ activates the expression of p75NTR in the AD brain, and the upregulated p75NTR, in turn, promotes Aβ production, thus forming a vicious cycle and finally resulting in Aβ over‐production.^9^, ^29^ Meanwhile, the extracellular domain of p75NTR (p75ECD), which is mainly generated by cleavage of tumor necrosis factor α converting enzyme (TACE, also known as ADAM17),^30^, ^31^ is reported to be a protective molecule for AD. Both intraventricular and muscular delivery of AAV‐p75ECD can exert preventive and protective effects on AD‐related pathology in vivo.^32^, ^33^ However, the level of p75ECD was decreased in the brain of AD patients, indicating the shedding of p75ECD is impaired in AD.^32^, ^34^ All evidence above suggests the important role of p75NTR/p75ECD imbalance in the pathogenesis and development of AD, rendering *NGFR* as a candidate gene in the study of AD genetic background.

The findings of our study show that rs2072446 is associated with an increased risk (OR = 1.79) of AD in the Chinese Han population. In 2008, the first study on the association between *NGFR* polymorphism and the risk of AD was conducted by Cozza et al.^12^ in the Italian population. In their study, only four SNPs in the *NGFR* gene were selected, and rs2072446 was also shown to be associated with an increased risk of “familial AD” (defined as those with at least two first‐degree relatives in two generations with AD, and no mutations in *APP*, *PSEN1*, and *PSEN2* genes) in the codominant genetic model (C/T vs. C/T, OR = 3.01, 95% CI = 1.00–9.12, *p* = 0.016). However, this association did not retain after multiple corrections. In the second relevant study conducted in Taiwan, China, only five SNPs (including rs2072446) were included, and the result showed no association between *NGFR* polymorphism and the risk of AD.^13^ A previous study also reported the association between rs9908324, another SNP in *NGFR*, and Aβ deposition, indicating a link between APP processing/Aβ accumulation and NGF/NGF receptor mediated signaling pathways.^11^ However, no association between rs9908324 and the risk of AD was found in our study. The discrepancies between the present study and previous studies may be explained by different ethnicities and sample sizes.

Another important finding of our study is that the minor allele (T) of rs2072446 is associated with the increased Aβ deposition, reflected by higher levels of AV45 uptake and lower levels of CSF Aβ42, in the brains of *APOE ε4* non‐carrying participants from the ADNI database. Besides, it is also shown to be associated with a shorter estimated time and an increased risk of AD progression in *APOE ε4* non‐carriers, which may be mainly attributed to higher levels of baseline Aβ burden. This can be considered equivalent to the results of subgroup analysis in the study conducted in Taiwan, which revealed that rs2072446 was associated with an increased risk of sAD in *APOE ε4* non‐carriers (OR = 2.18, 95% CI = 1.19–4.00, *p* = 0.007).^13^ The reason why *APOE ε4* status can undermine the association of rs2072446 with the risk of AD and Aβ accumulation may be explained as follows. First, *APOE ε4* is currently the most acknowledged genetic risk factor for sAD. It is shown that carrying one and two *APOE ε4* alleles can increase the risk of sAD by 3‐ to 4‐fold and 9‐ to 15‐fold, respectively,^35^, ^36^, ^37^ which are much higher than that of rs2072446 (OR = 1.79) after adjustment for age, sex, and *APOE* status in our study. Therefore, it is considered that *APOE ε4* may conceal the underlying association between rs2072446 and the risk of AD. Secondly, *APOE ε4* is consistently associated with greater Aβ deposition in the brain of cognitively healthy elderly individuals, and patients with AD and MCI.^38^, ^39^, ^40^ It is also associated with an increased rate of longitudinal Aβ accumulation among cognitively healthy individuals who are amyloid negative.^38^ It is also possible that *APOE ε4* may overshadow the effect of rs2072446 on Aβ deposition in the brain as well as subsequent disease progression. These findings indicate that more attention should be paid to the impact of *APOE ε4* status on the studies of AD candidate genes and biomarkers in the future.

As mentioned above, the levels of p75ECD are decreased in AD patients, and restoring the level of p75ECD can reduce the Aβ burden and other AD‐related pathology in transgenic AD mice.^32^ However, the regulatory mechanism of the impaired p75ECD shedding in AD remains unclear. The sequences of p75NTR are highly conserved among various species, while rs2072446, which leads to an amino acid change from serine to leucine at the 205th position (S205L) of p75NTR protein, is predicted to be not conserved and may affect the structure and function of p75NTR. Since the location of S205L is adjacent to the cleavage site of TACE, we speculate that this missense mutation may impair p75ECD shedding. This is indirectly supported by the fact that rs2072446 was indeed associated with the aggravated Aβ burden in the ADNI cohort, which might be due to lower levels of p75ECD in these minor allele (T) carriers. Unfortunately, only five CSF samples were obtained from the included subjects carrying S205L, which was inadequate to determine whether the level of p75ECD is deceased among them. Further studies are needed to reveal the biological function of rs2072446 (S205L), as well as other factors that may affect p75ECD shedding.

There are several limitations in our study. First, the AD patients included were diagnosed clinically without any evidence of CSF biomarkers or amyloid PET imaging. Second, although at a type I error rate of 0.05, the statistical power to detect a relative risk of 1.8 or more compared with the control group was calculated to be 80.9% for rs2072446, the sample size may not be large enough. Third, due to the relatively small sample size of AD patients (*n* = 47) whose genotypes of rs2072446 could be obtained, the association analysis was not performed in the ADNI cohort. The association between rs2072446 and the risk of AD needs to be validated in the future.

In conclusion, rs2072446 is associated with the risk of AD in the Chinese population and is also correlated with a higher amyloid burden and an increased risk of AD progression in the *APOE ε4* non‐carriers from the ADNI cohort. Our study reveals the role of p75NTR in AD from a genetic perspective and provides preliminary evidence of the effect of rs2072446 on p75ECD shedding.

## AUTHOR CONTRIBUTIONS

CYH and ZTW were responsible for data curation, formal analysis, methodology, software, and writing of the original draft. YYS, AYS, HYL, DWC, GHZ, and CRT were responsible for participants’ enrollment, collection of blood samples, and DNA extraction. JTY was responsible for the supervision and data collection. FZ and YJW were responsible for the conceptualization, funding acquisition, project administration, supervision, reviewing, and editing of the draft. All authors read and approved the final manuscript.

## CONFLICT OF INTEREST

The authors declare that they have no conflict of interest, financial or otherwise.

## INFORMED CONSENT

Informed written consent was obtained for all participants.

## Supporting information


Tables S1–S5

## Data Availability

The data that support the findings of this study are available from the corresponding author upon reasonable request.
