# Supplementary material for: Association of rs2072446 in the NGFR gene with the risk of Alzheimer's disease and amyloid‐β deposition in the brain
Source: CNS Neurosci Ther. 2022 Sep 8;28(12):2218–29. doi: 10.1111/cns.13965 (PMC9627368; doi:10.1111/cns.13965)
Supplement: Supplementary file 1 — Tables S1–S5 [file CNS-28-2218-s001.doc]

**Supplementary Table 1. Product size and primers of SNPs within the *NGFR* and *APOE* genes.**

| SNP ID | PCR Product size (bp) | PCR primer sequence | Ligase reaction primer sequence |
| --- | --- | --- | --- |
| rs1804011 | 189 | **FP^a^**:  AGCTTTGCCTTCCACGCTGTCT  **RP^a^**:  ACTGGAGCCATGCCACTGATGT | **P1^b^**: TTCCGCGTTCGGACTGATATGCTGCTTCCCTCTGCCTGACC  **P2^b^**: TACGGTTATTCGGGCTCCTGTGCTGCTTCCCTCTGCCTGACA  **P3^b^**: CTCTCAGGCATGCCTGTGTGTTTTTTTTT |
| rs2072446 | 244 | **FP**:  GGTCCTCACTCCTGTGGCCTTT  **RP**: GCAGCCAGGATGGAGCAATAGA | **P1**: TTCCGCGTTCGGACTGATATGCTGGGGGCTGTGCTGTACG  **P2**: TACGGTTATTCGGGCTCCTGTGCTGGGGGCTGTGCTGTGCA  **P3**: AGCCCTCTGRG:GGTGTGGACTTTTTTT |
| rs2537706 | 278 | **FP**:  CAGGACCTGCCAAGGGCTTAGT  **RP**:  CCAGAGGCTGGAGACACAGCTC | **P1**: TCTCTCGGGTCAATTCGTCCTTCCGCCTCTGGTCTGGCCTCG  **P2**: TGTTCGTGGGCCGGATTAGTCCGCCTCTGGTCTGGCCTCA  **P3**: CCTTGCTACCCTCCCAGCCTTTTTTTT |
| rs2584665 | 416 | **FP**:  CCCAAGGCCTAGAGCAGTGTGT  **RP**:  TTTCCATGGTCCTGATCATTTAAGC | **P1**: TCTCTCGGGTCAATTCGTCCTTCCATTGCTGACTCCTCAGGCTGG  **P2**: TGTTCGTGGGCCGGATTAGTCCATTGCTGACTCCTCAGGCTGT  **P3**: TTACATCATCTCTTTTGGTCTCAGTTTTCTTTTTTTTTTTTTTTTTTT |
| rs3785931 | 180 | **FP**:  CTGCAGATGTTCCCAGCACTGA  **RP**:  GGTTTGGTGGGGAAGGAAACAG | **P1**:  TCTCTCGGGTCAATTCGTCCTTAGCAGGACAGGCACAGGAAG  **P2**:  TGTTCGTGGGCCGGATTAGTAGCAGGACAGGCACAGGGAA  **P3**:  CAGAGGAGGTGGAACCTTGGTGTTTTTTTTTTT |
| rs534561 | 278 | **FP**: CAGGACCTGCCAAGGGCTTAGT  **RP**:  CCAGAGGCTGGAGACACAGCTC | **P1**:  TTCCGCGTTCGGACTGATATGCTACCCTCCCAGCCTTACG  **P2**:  TACGGTTATTCGGGCTCCTGTGCTACCCTCCCAGCCTTACC  **P3**:  CTACCATGCTCTGATCTYGGTGATTTTTTTTTTTTTTTT |
| rs603769 | 416 | **FP**:  CCCAAGGCCTAGAGCAGTGTGT  **RP**:  TTTCCATGGTCCTGATCATTTAAGC | **P1**:  TTCCGCGTTCGGACTGATATGATCAAATGAAGTAACGTGGTGTTCATGAC  **P2**:  TACGGTTATTCGGGCTCCTGTGATCAAATGAAGTAACGTGGTGTTCATGAT  **P3**:  AAGGAGGAACTCACTTCCCTTGCTTTTTTTTTTTTTTTTTTTTT |
| rs7219709 | 189 | **FP**:  AGCTTTGCCTTCCACGCTGTCT  **RP**:  ACTGGAGCCATGCCACTGATGT | **P1**:  TCTCTCGGGTCAATTCGTCCTTCGTGGAGATGGGATGCTTTCC  **P2**:  TGTTCGTGGGCCGGATTAGTCGTGGAGATGGGATGCTTCCT  **P3**:  TAGGRC:CTGGTCCATGATGGTTTTTTTTT |
| rs734194 | 248 | **FP**:  AAGCATCGGAGGGAATTGAGGT  **RP**:  CCCCACAGGTCACAGTCGAAGT | **P1**:  TTCCGCGTTCGGACTGATATGGCTGGAGCTGGCGTCTGACG  **P2**:  TACGGTTATTCGGGCTCCTGTGGCTGGAGCTGGCGTCTGACT  **P3**:  TCAAGGGCTTACAYGTGGAGGTTTTTTTTTTTTTT |
| rs741072 | 248 | **FP**:  AAGCATCGGAGGGAATTGAGGT  **RP**:  CCCCACAGGTCACAGTCGAAGT | **P1**:  TCTCTCGGGTCAATTCGTCCTTCGCATTCCCACACTGGCACG  **P2**:  TGTTCGTGGGCCGGATTAGTCGCATTCCCACACTGGCACA  **P3**:  CTTTTCWTCATCRCAACCTTCTGGGTTTTTTTTTTTTTTTTT |
| rs741073 | 266 | **FP**:  TTTTTCCTGAGCTTGGCCAGAA  **RP**:  GGCCAGCAGGTGAGTTTCCTC | **P1**:  TCTCTCGGGTCAATTCGTCCTTCTRT:TCTGTTTTGCCTGAAGTTGTAG  **P2**:  TGTTCGTGGGCCGGATTAGTCTRTTCTGTTTTGCCTGAAGTTGCAA  **P3**:  TGAGTGTGGCTCCCCTMTATTTAGCTTTTTTTTTTTTTTTTT |
| rs9908234 | 202 | **FP**:  TGCCTTTCTACCAAGGYGAGTTC  **RP**:  TCTGGGCTGCAATACAGGGAAA | **P1**:  TCTCTCGGGTCAATTCGTCCTTGGGAAGGGTTGCTTCAGGTCTCG  **P2**:  TGTTCGTGGGCCGGATTAGTGGGAAGGGTTGCTTCAGGTCTCA  **P3**:  CTCTGGTAGCAGCAAGAAGTGACATTTTTTTTTTTTTTTTTT |
| rs429358 | 317 | **FP**: AGGGCGCTGATGGACGAGAC  **RP**: GCCCCGGCCTGGTACACT |  |
| rs7412 | 185 | **FP**:  GGCGCGGACATGGAGGAC  **RP**: GCCCCGGCCTGGTACACT |  |

^a^ FR, forward primer. FR, reverse primer. **^b^** P1 and P2 indicates the allele-specific 5’ ligase primers for each SNP, and P3 indicates the 3’ ligase primer for each SNP.

**Supplementary Table 2. Characteristics of the study population from the Chongqing cohort.**

| **Characteristic** | **Total**  **(n = 756)** | **Control group**  **(n = 390)** | **sAD group**  **(n = 366)** | ***P* value** |
| --- | --- | --- | --- | --- |
| Age (years, mean ± SD) | 69.37 ± 9.33 | 68.89 ± 8.92 | 69.89 ± 9.67 | 0.077 |
| Sex (female, %) | 388 (51.32) | 191 (48.97) | 197 (53.82) | 0.18 |
| *APOE ε4* carriers (n, %) | 222 (29.37) | 83 (21.28) | 139 (38.00) | <0.001 |
| MMSE score (mean ± SD) | 20.83 ± 8.48 | 27.06 ± 3.36 | 14.04 ± 7.05 | <0.001 |
| CDR score (mean ± SD) | 0.91 ± 1.11 | 0 ± 0 | 1.90 ± 0.83 | <0.001 |
| CSF Aβ42 (pg/ml) ^†^ | 1062.62 ± 519.57 | 1385.05 ± 401.47 | 623.82 ± 289.42 | <0.001 |
| CSF t-tau (pg/ml) ^‡^ | 134.20 ± 78.00 | 109.44 ± 49.25 | 154.68 ± 90.63 | <0.001 |
| Comparison between the sAD group and control group in continuous variables was performed using *t-tests* and *χ^2^ tests* respectively. †, n=181 for control group and n=133 for sAD group; ‡, n=129 for control group and n=156 for sAD group. Abbreviations: sAD, sporadic Alzheimer’s disease; SD, standard deviation; *APOE*, apolipoprotein E gene; MMSE, Mini-Mental State Examination; CDR, Clinical Dementia Rating; Aβ, amyloid-beta; CSF, cerebrospinal fluid; t-tau, total tau; NA, not available. | | | | |

**Supplementary Table 3. Information of 12 tag-SNPs and HWE test.**

| Gene | tag-SNP | Location ^a^ | Allele (major/minor) | MAF in CHB ^b^ | MAF in Controls | *P* value of HWE |
| --- | --- | --- | --- | --- | --- | --- |
| NGFR | rs603769 | promotor | A/G | 0.208 | 0.221 | 0.66 |
| NGFR | rs2584665 | promoter | A/C | 0.128 | 0.150 | 0.33 |
| NGFR | rs9908234 | intron1 | A/G | 0.263 | 0.279 | 0.53 |
| NGFR | rs3785931 | intron1 | C/T | 0.500 | 0.424 | 0.76 |
| NGFR | rs2537706 | intron3 | G/A | 0.122 | 0.156 | 1.00 |
| NGFR | rs534561 | intron3 | C/G | 0.344 | 0.308 | 0.41 |
| NGFR | rs2072446 | exon4 | C/T | 0.148 | 0.077 | 1.00 |
| NGFR | rs7219709 | exon6 | C/T | 0.136 | 0.081 | 0.30 |
| NGFR | rs1804011 | exon6 | C/A | 0.135 | 0.123 | 0.63 |
| NGFR | rs734194 | exon6 | T/G | 0.186 | 0.308 | 0.91 |
| NGFR | rs741072 | exon6 | C/T | 0.408 | 0.371 | 0.27 |
| NGFR | rs741073 | exon6 | G/A | 0.250 | 0.241 | 1.00 |

**^a^ The genomic coordinates were shown based on the human assembly GRCh37, and relative locations were referred to the *NGFR* gene. ^b^ Data were obtained from HapMap database for Chinese Han in Beijing (CHB, n=45). Abbreviations: MAF, minor allele frequency; HWE, Hardy-Weinberg equilibrium.**

| **Supplementary Table 4. Associations of rs2072446 with indicators of amyloid deposition.** | | | | | | | | | | | |
| --- | --- | --- | --- | --- | --- | --- | --- | --- | --- | --- | --- |
| **SNP (Genotype)** | **AV45** | | |  | **CSF Aβ42 (pg/ml)** | | |  | **Plasma Aβ42 (pg/ml)** | | |
|  | **n** | **Mean ± SD** | **B, *P* value** |  | **n** | **Mean ± SD** | **B, *P* value** |  | **n** | **Mean ± SD** | **B, *P* value** |
| rs2072446 |  |  |  |  |  |  |  |  |  |  |  |
| CC | 467 | 1.19 ± 0.22 | 0.2234, 0.0721 |  | 561 | 1071.19 ± 464.64 | -0.2066, 0.0884 |  | 254 | 37.26 ± 12.13 | 0.0796, 0.7130 |
| TT/CT | 54 | 1.25 ± 0.26 |  |  | 56 | 956.92 ± 401.39 |  |  | 22 | 37.99 ± 12.13 |  |
| Adjusted for age, sex, *APOE ε4* status, clinical diagnosis, and ethnic category.  Abbreviations: SNP, Single nucleotide polymorphism; SD, standard deviation; CSF, cerebrospinal fluid; AD, Alzheimer’s disease; AV45, ^18^F-AV45 amyloid-PET; Aβ, amyloid-beta. | | | | | | | | | | | |

**Supplementary Table 5. Moderating effects of age, sex, and *APOE ε4* status on the association between rs2072446 and other AD endophenotypes**

| **Variable or interaction** | **CSF tau** | |  | **CSF P-tau** | |  | **Entorhinal cortex/ICV** | |  | **Hippocampus/ICV** | |  | **Whole Brain/ICV** | |
| --- | --- | --- | --- | --- | --- | --- | --- | --- | --- | --- | --- | --- | --- | --- |
|  | **B** | ***P* value** |  | **B** | ***P* value** |  | **B** | ***P* value** |  | **B** | ***P* value** |  | **B** | ***P* value** |
| **Model for age effect** |  |  |  |  |  |  |  |  |  |  |  |  |  |  |
| rs2072446^TT/CT^ | -0.8496 | 0.5035 |  | -0.9339 | 0.4565 |  | -0.5197 | 0.6654 |  | -0.6705 | 0.5299 |  | -0.8927 | 0.4073 |
| rs2072446^TT/CT^ × age | 0.0121 | 0.4833 |  | 0.0135 | 0.4305 |  | 0.0092 | 0.5707 |  | 0.0104 | 0.4712 |  | 0.0122 | 0.4003 |
| **Model for sex effect** |  |  |  |  |  |  |  |  |  |  |  |  |  |  |
| rs2072446^TT/CT^ | 0.3961 | 0.3234 |  | 0.2917 | 0.4616 |  | -0.0773 | 0.8378 |  | 0.1206 | 0.7234 |  | -0.6109 | 0.0695 |
| rs2072446^TT/CT^ × sex | -0.2461 | 0.3432 |  | -0.1653 | 0.5193 |  | 0.1552 | 0.5110 |  | -0.0166 | 0.9371 |  | 0.4068 | 0.0527 |
| **Model for *APOE ε4* effect** |  |  |  |  |  |  |  |  |  |  |  |  |  |  |
| rs2072446^TT/CT^ | 0.0803 | 0.6326 |  | 0.1082 | 0.5145 |  | 0.1512 | 0.3382 |  | -0.0236 | 0.8634 |  | 0.0350 | 0.8024 |
| rs2072446^TT/CT^ ×*APOE ε4* | -0.1080 | 0.6820 |  | -0.1422 | 0.5849 |  | 0.0167 | 0.9439 |  | 0.2808 | 0.1861 |  | -0.0590 | 0.7802 |
| Adjusted for age, sex, *APOE ε4* status, clinical diagnosis, and ethnic category. Abbreviations: CSF, cerebrospinal fluid; P-tau, hyperphosphorylated tau; *APOE*, apolipoprotein E gene; ICV, intracranial volume. | | | | | | | | | | | | | | |
